# Noncoding RNA (ncRNA) Profile Association with Patient Outcome in Epithelial Ovarian Cancer Cases

**DOI:** 10.1007/s43032-020-00372-7

**Published:** 2020-10-30

**Authors:** Douglas V. N. P. Oliveira, Kira P. Prahm, Ib J. Christensen, Anker Hansen, Claus K. Høgdall, Estrid V. Høgdall

**Affiliations:** 1 Department of Pathology, Herlev Hospital, University of Copenhagen, Herlev, Denmark; 2 Oncology Venture, Hoersholm, Denmark; 3 Department of Gynecology, Juliane Marie Centre, Rigshospitalet, University of Copenhagen, Copenhagen, Denmark

**Keywords:** ncRNA profiling, Biomarker, Ovarian cancer, High throughput

## Abstract

Ovarian cancer (OC) is the second most frequent type of gynecological cancers worldwide. In the past decades, the development of novel diagnostic and prognostic biomarkers available for OC has been limited, reflecting by the lack of specificity of such markers or very costly management. Microarray expression profiling has shown very effective results in exploring new molecular markers for patients with OC. Nonetheless, most screenings are focused on mutations or expression of molecules that are translated into proteins, corresponding to only 2% of the total human genome. In order to account for the vast majority of transcripts, in the present exploratory study, we assessed the expression levels of a comprehensive panel of noncoding RNA in different subtypes of OC. We further evaluated their association with patient overall survival (OS) and aggressive forms of the disease, such as tumor type, stage, and chemotherapy resistance. By microarray profiling in a total of 197 epithelial OC patients (162 serous carcinomas, 15 endometrioid carcinomas, 11 mucinous carcinomas, and 9 clear cell carcinomas), we found two candidates, SNORA68 and SNORD74, which associated with OS and poor clinicopathological features. The overexpression of those two targets combined was correlated with shorter OS and progression-free survival. That association was further observed to correlate with a more aggressive form of the disease. Overall, the results indicate that a panel comprised of SNORA68 and SNORD74 may be clinically relevant, where patients could be offered a more individualized, targeted follow-up, given its further validation on future prospective clinical studies.

**Supplementary Information:**

The online version contains supplementary material available at 10.1007/s43032-020-00372-7.

## Introduction

Globally, ovarian cancer (OC) is the eighth most common cancer for women and the second most common cause of gynecologic-associated cancer death [1, 2]. Within the past 25 years, all cancer collectively have presented an improvement in overall survival (OS) rate by 27% in developed countries, with an exception of OC [2, 3]. Survival rate increment for OC has been marginal worldwide, with a 5-year survival remaining at approximately 41–47%, and with an estimate of approximately 293,000 new cases and 185,000 associated deaths annually [1, 2]. The outcomes of OC are dismal, given its asymptomatic characteristics and distinct subtypes, each with its own biological and molecular features, leading to 65% of all OC cases being diagnosed at an advanced stage (International Federation of Gynecology and Obstetrics, FIGO III–IV), and a 5-year OS ranging from 30 to 50% [4]. In Denmark, cases diagnosed at a later stage account for 75%, while median OS is at 15.6 months [5]. Improvements on the treatment guidelines have also been limited, where cytoreductive surgery followed by adjuvant platinum-based chemotherapy remains the current standard therapy of OC patients. Furthermore, the etiology of this disease is still unclear, reflecting the heterogeneity of OC and its distinct subtypes. Currently, CA-125 is a widely used biomarker for risk stratification in OC management, presented in high levels in approximately 80% of epithelial OC (EOC) cases [6]. Nonetheless, this biomarker is relatively unspecific, as its levels are also associated with menstruation, endometriosis, or other inflammatory diseases. In order to change the current scenario, the discovery and use of new biomarkers are crucial for better management of OC, such as prediction of OS, progression of the disease, and response to treatment.

Genome sequencing and gene expression profiling have been the methods of choice in clinical studies screening for potential molecular biomarkers. These methods provide tumor molecular information, which can contribute to improve diagnosis and outcome, such as prediction of chemotherapy response and impact on patient survival [7–10]. The practical reason is that they give a straightforward dimension of mutations on the genome that alter protein-coding messenger RNA (mRNA) genes. Nonetheless, this coding fraction of RNA accounts for less than 2% of the whole transcribed human genome, where the remaining majority are noncoding RNA (ncRNA) [11, 12]. Noncoding RNAs are small molecules that recently have been found to have regulatory and infrastructural roles in the maintenance of cell, such as microRNA (miRNA), small interfering RNA (siRNA), long noncoding RNA (lncRNA), enhancer RNA (eRNA), and small nucleolar RNA (snoRNA). In OC, the role of ncRNAs is vastly illusive, with limited studies focused on the role of subgroups of ncRNA, such as miRNA and lncRNA expression [13–16]. A metadata analysis has recently found the association of lncRNA with OS and progression-free survival (PFS) [16]. Previously, our group has shown that a selective panel of miRNAs was able to distinguish late-stage OC patients from those with a benign pelvic mass [13]. Thus, the identification of ncRNA in OC poses a critical challenge in determining the association of such elements with the outcome of the disease, with potential hitherto unexplored.

On this exploratory study, we investigated the association of ncRNAs across patients with different subtypes of EOC with OS, PFS, tumor type, grade, and resistance to chemotherapy. Despite the particular clinical characteristics of each subtype, we aimed to establish a mutual molecular feature across them which equally affects patient outcome. We observed that a panel of candidate snoRNAs was associated with OS and PFS. We further observed that the combination of such candidates and classification into different categories did also correlate with other clinicopathological characteristics associated to poor prognosis.

## Materials and Methods

### Patients and Samples Collection

All tissue samples were obtained from patients enrolled in the Danish Pelvic Mass study, a national ongoing cohort initiated in 2004. Patients were diagnosed and surgically treated for EOC between October 2004 and January 2010. The exclusion criteria were non-epithelial OC, neoadjuvant chemotherapy, scarce tissue material for analysis, and a history of another cancer type. Subjects that had received primary cytoreductive surgery with the confirmation of an epithelial histologic subtype were included in this study. All patients were registered in the Danish Gynecologic Cancer Database (DGCD) [17], a national mandatory clinical database, as well as in Bio- and Genome Bank, Denmark (RBGB, www.regioner.dk), a registry including clinical biobanks, ensuring biological material of high quality for patients own treatment and biomarker research. The study was carried out according to the guidelines of the Declaration of Helsinki, including written informed consent from all subjects, and it has been approved by the Danish National Committee for Research Ethics, Capital Region (approval codes KF01-227/03 and KF01-143/04). All patients were followed until either death of any cause, emigration or January 17, 2015.

Tumor tissues were stored as formalin-fixed and paraffin-embedded (FFPE), and all samples were registered in RBGB/Danish Cancer Biobank. A specialized pathologist in gynecology has revised histologic diagnosis for all tissue samples. With conventional hematoxylin and eosin staining, all samples included presented a tumor presence above 50%.

### RNA Extraction and ncRNA Profiling

Total RNA was extracted from 20-μm-thick FFPE tumor sections using the RecoverAll Total Nucleic Acid Isolation Kit for FFPE samples (Ambion, USA). Samples were then hybridized to Affymetrix GeneChip miRNA 2.0 Array (Affymetrix, USA), following the manufacturer instructions. Briefly, RNA samples were subjected to two cycles of cDNA conversion, amplified, and labeled with biotinylated ribonucleotide analogues, generating cRNA single strands. Synthesized strands were then purified, heat-induced fragmented, and finally hybridized to the microarray chip. Microarrays were scanned in a GeneChip Scanner (Affymetrix, USA), and data acquisition was performed by GeneChip Command Console (Affymetrix, USA).

### Data Treatment and Statistical Analysis

Raw data were background-corrected, normalized, and log-transformed by applying the robust multi-array average (RMA) method [18], resulting in a total of 909 probes. This method performs raw intensity values adjustment by utilizing background correction, log_2_ transformation, followed by quantile normalization. Normalized data were primarily submitted to Cox univariate regression, identifying 102 probes (*P* < 0.05). Due to the large number of predictors, a LASSO-penalized model for Cox multivariate regression was applied, and the resulting targets were finally cross-validated (10-fold) by a last round of Cox multivariate analysis. The primary outcome for the investigation of candidate biomarkers was OS, defined as time in months, counting from the time of diagnosis to time of death, or last censored follow-up. The association between the candidate targets and the clinicopathological features of the patients were investigated by multivariate logistic regression model, considering all discovered targets combined for each feature alone. The clinical characteristics assessed were tumor type, stage, progression of disease, resistance to first line of chemotherapy, and menopause status. All analyses with the clinical features were adjusted for age and CA-125 levels. In the latter case, CA-125 was included in the analysis given its use in risk estimation of malignancy of ovarian disease. Receiver operating characteristic (ROC) curves were calculated in order to assess the efficiency of prediction of our models presented by the area under the curve (AUC). Targets with *P* < 0.01 were considered associated with patient survival, and a penalty alpha = 1.0 was applied for the LASSO method. All statistical analyses were performed in the R environment (https://www.R-project.org/).

### Independent Cohort Assessment

Normal ovarian epithelium and OC samples from independent cohorts were used in order to assess transcription expression of the candidate markers. The first cohort was comprised by 6 normal and 32 OC tissue samples [19]. The second cohort contained 10 normal and 53 OC samples [20]. Both cohorts were retrieved from the Gene Expression Omnibus (GEO) database, with their corresponding identification, GSE40595 and GSE18521, respectively. Raw data were background-corrected, normalized, and log-transformed by applying the RMA method.

## Results

### Clinical and Pathological Features of the Patients

Primarily, 246 patients with EOC were identified and included. From those, 49 subjects were excluded due to either insufficient tumor material for analysis (*n* = 24), neoadjuvant chemotherapy or ongoing palliative care (*n* = 15), other forms of cancer (*n* = 8), or ambiguous histologic classification (*n* = 2). A total of 197 patients were eligible for inclusion in the study. Histological subtypes were represented by 162 (82.2%) serous adenocarcinomas, 15 (7.6%) endometrioid adenocarcinomas, 11 (5.6%) mucinous adenocarcinomas, and 9 (4.6%) clear cell carcinomas. Early-stage diagnoses (FIGO I–II) accounted for 52 (26.4%) of the cases, while 145 (73.6%) were advanced stage (FIGO III–IV). Low-grade tumors were found in 20 (10.2%) patients and high grade in the remaining 177 (89.8%) subjects. Thirty-nine (19.8%) subjects were categorized with type I tumor and 158 (80.2%) with type II tumor. Briefly, type I tumors develop from benign extraovarian lesions that implant on the ovary and which can subsequently undergo malignant transformation, whereas most of type II carcinomas develop from intraepithelial carcinomas in the fallopian tube [21]. In terms of tumor behavior, the latter are generally more aggressive. Patient demographics are shown in Table 1.

**Table 1:** Clinicopathological characteristics of OC patients

Status	
Alive	64 (32.5%)
Death	133 (67.5%)
Median age in years (range)	64 (31–89)
Median OS^(1)^ in months	48 (95% CI: 40–52)
Histology	
Serous adenocarcinoma	162 (82%)
Mucinous adenocarcinoma	11 (6%)
Endometrioid adenocarcinoma	15 (8%)
Clear Cell adenocarcinoma	9 (4%)
FIGO stage^(2)^	
IA	22 (11.2%)
IB	2 (1.0%)
IC	7 (3.6%)
IIA	3 (1.5%)
IIB	6 (3.0%)
IIC	12 6.1%)
IIIA	3 (1.5%)
IIIB	10 (5.1%)
IIIC	106 (53.8%)
IV	26 (13.2%)
Histologic grade	
1	20 (10%)
2	102 (52%)
3	74 (38%)
Unknown	1 (< 1%)
Type I or II	
I	39 (19.8%)
II	158 (80.2%)

^(1)^*OS* overall survival

^(2)^*FIGO* International Federation of Gynecology and Obstetrics

### ncRNA Associated with Overall Survival

A panel comprised of 909 snoRNAs was analyzed as predictor variables in regard to OS of the patients. We started by examining the association of each target alone to OS. Therefore, each of the snoRNAs was submitted to univariate Cox regression analysis, and all discovered candidates were annotated. In total, 102 targets were found as potential predictors (*P* < 0.05) (Table S1). We proceeded by evaluating the combination of all predictors in association with OS, by multivariate Cox regression analysis. In this case, a lasso-penalty variation was implemented followed by cross-validation (10 iterations), resulting in 37 targets. We chose this method in order to improve the prediction of our model, by accounting for the large number of candidates primarily found and validate it by random iteration, respectively. From those potential candidates, a second round of analysis with all targets combined was performed in order to verify those predictors. Finally, for a better and robust clinical significance, only candidates with an absolute hazard ratio (HR) above 2.5 were considered (*P* < 0.01). In total, 2 targets were identified—SNORA68 (HR, 5.57; median expression, 1.17) and SNORD74 (HR, 2.63; median expression, 1.63), with their expression detected in all samples (Table 2). In summary, the overexpression of those snoRNAs showed a significant association with poor OS of patients. The analysis workflow employed is presented on Supplementary Fig. S1.

**Table 2:** Statistical summary of the discovered snoRNA

	Log2 expr.^(1)^	HR	95% CI	*P*
SNORA68	1.17	5.57	12.44	0.00004
SNORD74	1.63	2.63	4.92	0.00252

^(1)^Log_2_ expression as median values

### Overexpression of snoRNAs Predicts Poor OS

**Fig. 1:**
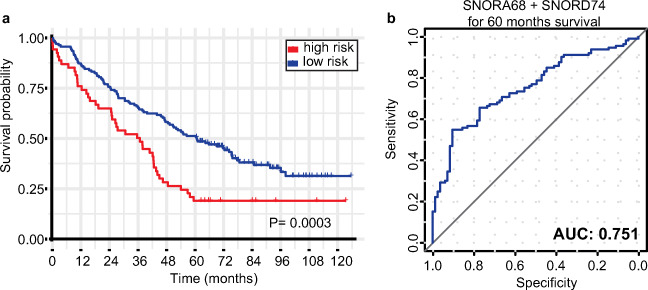
Overall survival and prediction performance of SNORA68 and SNORD74. **a** Survival curve shows that “high-risk” (both candidates overexpressed) group has a shorter survival compared to “low-risk” (at least one candidate downregulated) OC patients. **b** AUC/ROC curve of combination of SNORA68 and SNORD74 for 5-year OS. *P* value and AUC are presented above

Given the fact that the overexpression of SNORA68 and SNORD74 showed a positive association with shorter OS, we divided our patient cohort into 2 distinct subgroups: “high risk” and “low risk” for short survival. The “high risk” subgroup comprised of those patients whose expression level of both targets was overexpressed (above the median) (Supplementary Fig. S1). On the other hand, patients that did not fit the criteria were classified as “low risk.” Taking in consideration it was a supervised classification, we first evaluated the overall ncRNA expression profile on both groups, in order to ensure that the classification was not affected by marginal differences in expression values. We assessed the overall expression profile on both groups, which were significantly different (*P* < 0.0001) (Supplementary Fig. S2). Moreover, we sought to investigate the clinical implications of the overexpression of SNORA68 and SNORD74 on an unsupervised manner. The 5-year OS is generally used in the clinic as a means to observe the efficiency of a treatment, especially in more aggressive forms of the disease, where life expectancy is short. The classification showed distinction between subgroups, where patients whose both targets were overexpressed had a significantly lower OS in comparison to the rest, with 5-year OS of 18.5% and 51.0%, respectively (*P* = 0.0003) (Fig. 1a). Noteworthy, when SNORA68 and SNORD74 were examined individually, their expression levels presented inferior performance when compared to their combination, with 5-year OS of 33.3% (*P* = 0.035) and 31.3% (*P* = 0.028) for the overexpressed (above the median for each target) individuals, respectively (Supplementary Fig. S3). We further investigated the efficiency of both candidates to discriminate between patients with OS longer or shorter than 5 years by calculating the ROC curve, irrespective of their groups. The combination of both markers showed a predictive performance of AUC = 0.751 (Fig. 1b).

We further examined whether the over-representation of serous adenocarcinoma (162/197 samples) was responsible for our observations. To that end, we first performed a survival curve analysis in this group alone. The difference in OS was still observed in “high-risk” and “low-risk” subgroups (*P* < 0.0001) (Supplementary Fig. S4a). Next, we compared the survival curves between serous adenocarcinoma with all other subtypes combined. Given that the “high-risk” subgroup for non-serous samples was not sufficient for this comparison, we performed only in the “low-risk” subgroup. Results showed no difference between histologic subtypes of EOC (*P* = 0.35) (Supplementary Fig. S4b). Furthermore, the ROC curve performed poorly, close to baseline in distinguishing serous from all other subtypes (AUC = 0.582) (Supplementary Fig. S4c). No differences in expression levels were detected among all the histologic subtypes (Supplementary Fig. S5). These results indicate that rather being specifically associated with a subtype of EOC, the overexpression of SNOR68 and SNORD74 correlate with shorter patient survival in all histologic subtypes.

### High Expression of SNORA68 and SNORD74 Associate with Poor Prognosis

**Fig. 2:**
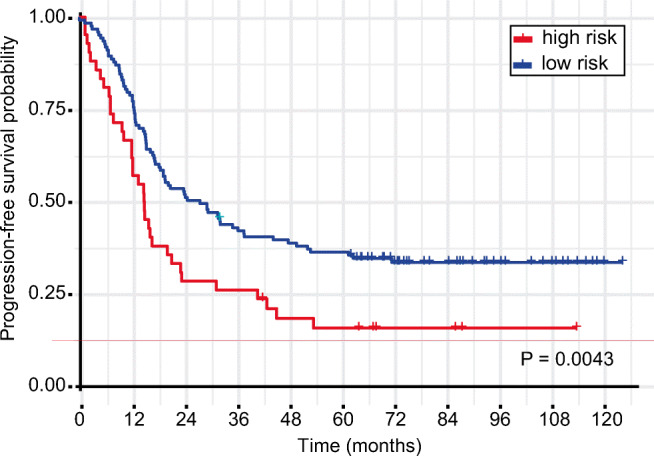
Progression-free survival for patients with overexpressed SNORA68 AND SNORD74. Survival curve shows that “high-risk” (all overexpressed candidates) groups have an overall shorter time to recurrence outcome compared to “low risk” (at least one candidate downregulated) in OC patients. *P* value is presented above

We also examined whether SNORA68 and SNORD74 would also be associated with patient PFS, by following the same classification in “high” and “low”-risk subgroups. In agreement with the observations from OS, the overexpression of these targets also showed an association with a shorter PFS compared to the other group, with 38.1% (16/42) and 21.1% (26/123) relapse of the disease within the first 12 months, respectively (*P* = 0.0043) (Fig. 2). Moreover, we tested whether the expression of the 2 targets interacted with the histologic subtype. In line with previous results, no interactions were observed between SNORA68 (*P* = 0.67) and SNORD74 (*P* = 0.96) with the subtype.

**Fig. 3:**
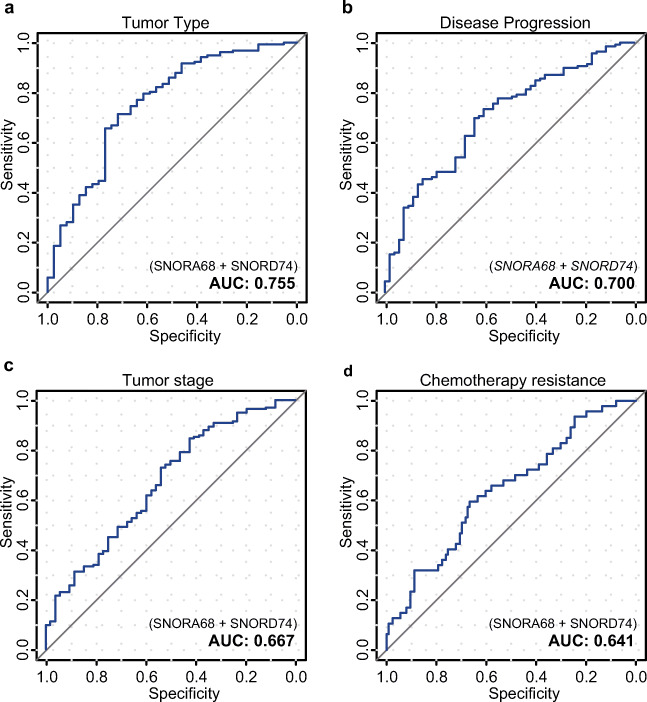
Prediction performance of SNORA68 and SNORD74 in clinicopathological outcome. AUC/ROC curves for the combination of SNORA68 and SNORD74 associated with **a** tumor type (types I and II), **b** disease progression (progression and no progression), **c** tumor stage (FIGO I–II and III–IV), and **d** chemotherapy response (no response, tumor recurrence within the first 6 months; and response, no tumor recurrence within the first 6 months). AUC values are presented above

In order to further investigate whether SNORA68 and SNORD74 can provide a clinical implication, we next investigated whether their combination is also able to predict clinicopathological features mostly associated with poor prognosis. To that end, we examined these snoRNAs as potential predictors for tumor type (I and II), stage (FIGO I–II and FIGO III–IV), disease progression (whether disease progressed until last follow-up), and primary platinum-based chemotherapy response (tumor recurrence within the first 6 months post-treatment and later), separately. We found that SNORA68 and SNORD74 were correlated with all these clinical characteristics. The overexpression of both targets showed association with tumor type II, with AUC of 0.755. It was also linked to shorter tumor progression (AUC = 0.700) and, to a lesser extent, to tumor stage (AUC = 0.667) and primary chemotherapy resistance (AUC = 0.641) (Fig. 3). None of the predictors was shown to be associated with menopause status (Table 3).

**Table 3:** Association of candidates with clinicopathological characteristics

	SNORA68		SNORD74	
	OR	95% CI	OR	95% CI
Tumor type (I and II)	3.06	0.62–16.50	1.27	0.38–4.46
Tumor stage (FIGO I–II and III–IV)	5.71	1.34–27.01	1.45	0.51–4.28
Disease progression	6.86	1.62–32.82	1.41	0.49–4.26
Chemotherapy resistance	0.80	0.18–3.39	2.49	0.91–7.03
Menopause	0.76	0.17–3.30	0.99	0.33–2.92

*OR* odds ratio, *CI* confidence interval

Overall, our findings indicate that SNORA68 and SNORD74 performed well in predicting poor OS for EOC patients, and rather specifically to serous adenocarcinoma, they were found in all histologic subtypes examined.

Considering that the high expression of both SNORA68 and SNORD74 associated with poor prognosis, we further speculated that their expression levels would be lower in normal ovarian tissues. Thus, we interrogated their expressions on 2 independent cohorts containing normal and malignant ovarian tissues, deposited on GEO database, namely, GSE40595 and GSE18521 [19, 20]. Noteworthy, no OC studies performed on the same chip array as our current were found. Hence, we chose those studies based on the same platform, so the same normalization method could be applied. Neither contained information about SNORD74. Nonetheless, on both cohorts, we observed that the expression of SNORA68 was higher in OC cases compared with normal ovarian tissue (*P* = 0.00008 and *P* = 0.039, respectively) (Supplementary Fig. S6).

## Discussion

Considering that approximately 65% of all OC cases are diagnosed at an advanced stage (FIGO III–IV), the discovery of novel biomarkers diagnosing OC at an early asymptomatic stage or/and being useful in prediction of effective personalized treatment could be beneficial in order to improve the prognosis of patients and avoid complications based on ineffective traditional therapies. Currently, CA-125 protein measurement is the most accepted and employed marker for management of the disease, despite its limited specificity to OC. Early studies showed that its level fluctuates in association with other conditions, such as inflammation, menstruation, endometriosis, and other non-ovarian malignancies [22–24]. Hence, discovery of new molecular biomarkers still presents a clinical challenge with unmet needs. In that regard, there has been a considerable amount of studies focused on unraveling biomarkers based on molecules with known functional role, such as DNA mutation status and mRNA profiling of patients. In the case of mRNA, this fraction corresponds to less than 2% of the whole transcribed human genome, where the great majority is noncoding RNA [11, 12]. These molecules encompass a large category of RNA species, including microRNAs, small nucleolar RNAs, short-interfering RNAs, Piwi-associated RNAs, small Cajal body-specific RNAs, and small nuclear RNAs. Relatively recent studies have demonstrated their crucial roles in a range of regulatory processes, such as organism development and pathogenesis [25, 26]. Among the subfamilies of ncRNA, miRNA are the most extensively investigated, given the fact that they were shown to act as transcription regulators of many cancer-associated genes [13, 27]. However, snoRNA, together with their host genes, may also play a critical role in cancer development [28]. Similar diversity and number of snoRNAs compares to those of miRNA, further highlighting these molecules as potential novel molecular class with significant influence on tumor progression. Here, in a cohort of patients with EOC, we investigated the expression profile of ncRNA based on patient OS. We further extended the analysis of the candidate biomarkers to clinicopathologic characteristics associated with poor prognostics.

In OC, this field of investigation is quite recent and mostly limited to long noncoding RNA (lncRNA), emphasizing the role of ncRNA as emerging novel biomarkers [14, 29, 30]. One group has performed a preliminary ncRNA screening in OC patients and found 235 targets differentially expressed. From those, they further validated four targets—MIAT, SNORD114, SNORD114-2, SNORD114-10, and SNORD114-11—in matched omental metastasis. Moreover, there has been no investigation of whether snoRNA associates with OS of OC patients or other clinicopathological characteristics. In the present study, we sought to identify a distinctive pattern of dysregulated ncRNAs common across different subtypes of EOC, primarily in regard to OS of patients. Further evaluation was performed in order to verify whether the discovered candidate targets would also indicate a more severe set of clinicopathological features. We performed a comprehensive screening of ncRNAs by microarray technology on a cohort of 197 patients with EOC. For improved performance and sensitivity, the primarily discovered targets were submitted to a penalty-based scoring followed by a 10-fold cross validation, resulting in the elimination of weakly associated targets with OS or other targets. That resulted in the discovery of 2 snoRNAs: SNORA68 and SNORD74. They showed association with OS in all EOC subtypes rather than with one alone. The overexpression of both targets was associated with shorter OS. Those results were similar when observing PFS among patients.

Due to the complexity of OC, the survival of a patient is highly linked to various clinicopathological outcomes, such as tumor type, stage, the relapse of the disease, and sensitivity to chemotherapy [31–34]. Here, we found that the combination of both SNORA68 and SNORD74 was capable to clearly differentiate between (1) type I and II tumors; (2) progressive and stable disease; and, to a lesser extent, (3) tumor stage and (4) chemo-resistant and chemo-sensitive patients.

Given the novelty in evaluating snoRNA levels in OC, none of the targets have been previously reported. Interestingly, SNORA68 is located at the p13.1 locus on chromosome 19 (19p13.1), a region previously associated with susceptibility to both ovarian and breast cancer in the population with *BRCA1* or *BRCA2* mutation carriers [35, 36]. Bolton and colleagues reported two single nucleotide polymorphisms (SNPs) located on 19p13.1, rs8170, and rs2363956, associated with patient survival in a cohort of 8951 individuals [36]. Despite not showing evidence for that association on further validation, they observed that those markers were good predictors for susceptibility of all EOC, with better performance for the serous subtype. In the current study, we observed that the expression of SNORA68 shows similar patterns among all histologic subtypes analyzed. Despite not available in the present cohort, it would be interesting to investigate whether the expression of SNOR68 also differs between *BRCA1/2*-positive and *BRCA1/2*-negative cases. That could potentially represent a meaningful outcome in the clinical setting, where the expression of SNORA68 and *BRCA1/2* mutation status could be used in concert, in order to further predict patient response to PARP inhibitors [37, 38].

In the present study, SNORD74 was also found to be associated with patient OS and PFS. Furthermore, worse prognostic features, such as tumor type II, recurrence, and platinum-based chemotherapy resistance also showed positive correlation with overexpression of that molecule. It belongs to the box C/D snoRNA family, characterized by guiding the 2’-O-methylation of ribosomal RNA (rRNA) [39]. Some members of this family have been recently found to enhance the proliferation, migration, and invasion of tumors [40, 41]. For instance, SNORD126 was found highly expressed in colorectal cancer and hepatocellular carcinoma and further associated with tumor stage in the latter. They proceed to perform in vitro validation by inducing overexpression of that snoRNA in hepatocellular cell lines, which resulted in increased cell growth and chemoresistance, driven by activation of FGFR2-dependent PI3K-AKT signaling pathway [41]. Furthermore, SNORD74 is located in the intronic region of its host gene, *GAS5.* It has been reported to act as a tumor suppressor in different types of cancer [42–44]. Specifically in OC, *GAS5* was shown to induce apoptosis by promoting the expression of pro-apoptotic factors, such as Bcl2-associated X (*BAX*), Bcl-2 homologous antagonist killer (*BAK1*), and cleaved-caspase 3 and 9 [45]. By inducing cell proliferation, migration, and invasion, Li and colleagues further concluded that *GAS5* expression indicated poor prognosis in OC patients [44]. Finally, more recent studies indicated that the expression of *GAS5* might be regulated by microRNA-21, a ubiquitous miRNA associated with a plethora of cancer types. Noteworthy, in line with our findings, SNORA68 and SNORD74 have been reported highly expressed in lung cancer, with the former further validated on an independent cohort [46]. SNORA68 also presented a positive association with short OS of those patients, being suggested as a good predictive marker [46]. Noteworthy, we sought to validate our findings on independent cohorts from public databases, containing different OC histological subtypes. However, we could not find available datasets in EOC studies, further underscoring the exploratory and novelty aspects of the current results. On the other hand, we were able to investigate the expression levels of SNORA68 on normal ovarian and OC samples on two different cohorts. In both cases, data showed that the expression of SNORA68 was lower in normal tissues, indicating that its high expression might be associated with poor prognosis in OC cases.

In summary, we demonstrated that SNORA68 and SNORD74 performed well in predicting OS and PFS. We showed that their combination performed better than each candidate alone. That combination further associated with a more aggressive form of OC—shorter PFS, high-grade serous (type II) tumors, stage, and chemotherapy resistance. Moreover, considering that single biomarkers can be sensitive in predicting tumors at the cost of very low specificity, a panel of markers can provide a more accurate outcome. To that extent, we sought into determining ncRNA predictors that could associate with a shorter OS and further poor prognosis in patients with OC. Overall, this indicates that a panel comprised of a few predictors that associates with a more aggressive form of OC might be clinically relevant in planning treatment course, by presenting a better performance than a single marker. In that regard, potentially, a patient presenting overexpression of both SNORA68 and SNORD74 could be offered a more individualized, targeted follow-up. However, considering the novelty of snoRNAs as potential biomarkers, the current study aims at as an exploratory work, where further validation and functional analyses are necessary in order to unveil how they might regulate tumorigenesis or whether they are a consequence. Nonetheless, it allows for an excessive amount of approaches for future investigation, which can benefit in overall improvements for OC cancer patients.

## Supplementary Information

ESM 1 (PDF 869 kb)

ESM 2 (PDF 1030 kb)

ESM 3 (PDF 890 kb)

ESM 4 (PDF 894 kb)

ESM 5 (PDF 1006 kb)

ESM 6 (PDF 882 kb)

ESM 7 (XLSX 13 kb)
