# Supplementary material for: Noncoding RNA (ncRNA) Profile Association with Patient Outcome in Epithelial Ovarian Cancer Cases
Source: Reprod Sci. 2020 Oct 30;28(3):757–65. doi: 10.1007/s43032-020-00372-7 (PMC7862201; doi:10.1007/s43032-020-00372-7)
Supplement: Supplementary file 1 — (PDF 869 kb) [file 43032_2020_372_MOESM1_ESM.pdf]

S1 Figure

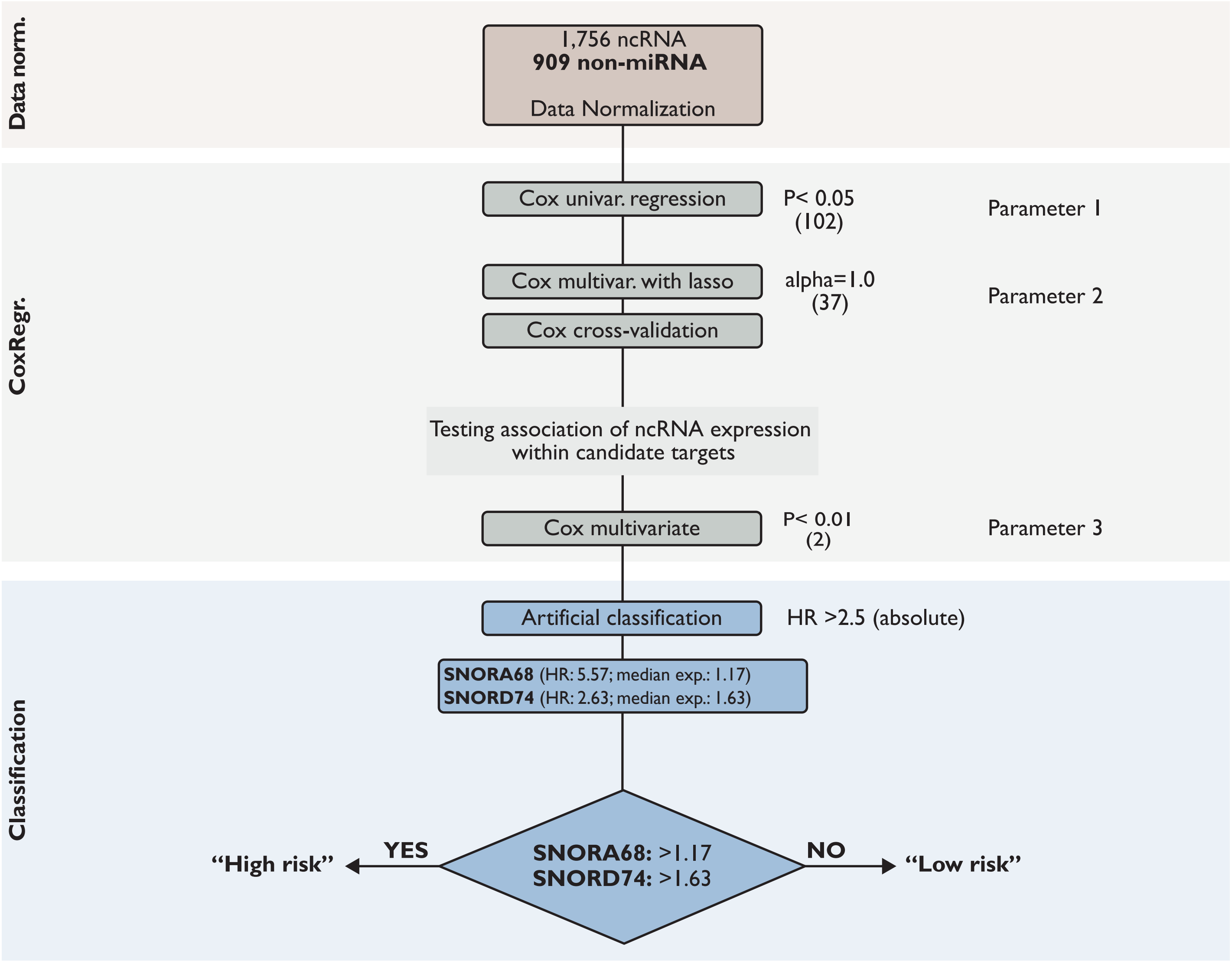

S1 Figure. Analysis workflow. "High risk" refers to the group of patients whom both SNORA68 and SNORD74 are overexpressed, whilst "Low risk" represents those patients that present only one or none of the markers overexpressed. The parameters (1, 2 and 3) indicate each step of the statistical analysis.
