# Supplementary material for: Noncoding RNA (ncRNA) Profile Association with Patient Outcome in Epithelial Ovarian Cancer Cases
Source: Reprod Sci. 2020 Oct 30;28(3):757–65. doi: 10.1007/s43032-020-00372-7 (PMC7862201; doi:10.1007/s43032-020-00372-7)
Supplement: Supplementary file 2 — (PDF 1030 kb) [file 43032_2020_372_MOESM2_ESM.pdf]

S2 Figure

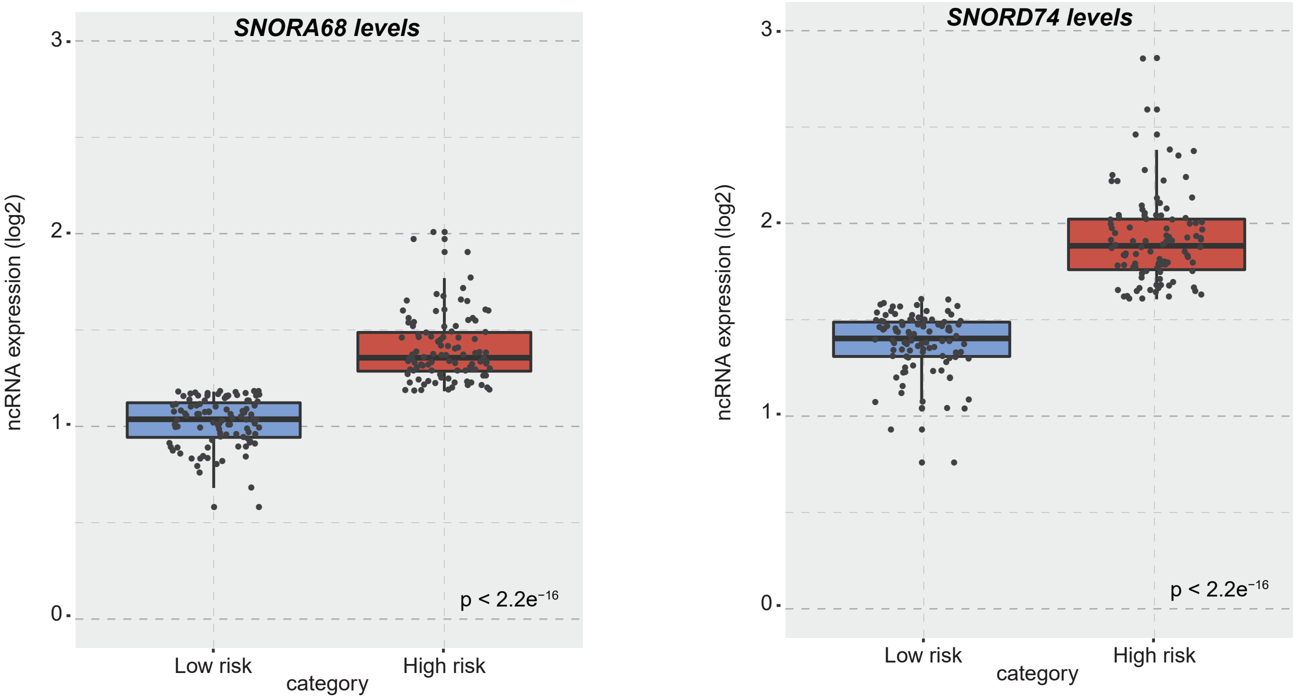

S2 Figure. ncRNA in "low risk" (blue) and "high risk" (red) groups. Values are normalized and provided as log_2 and P-value is presented above.
