# Supplementary material for: Noncoding RNA (ncRNA) Profile Association with Patient Outcome in Epithelial Ovarian Cancer Cases
Source: Reprod Sci. 2020 Oct 30;28(3):757–65. doi: 10.1007/s43032-020-00372-7 (PMC7862201; doi:10.1007/s43032-020-00372-7)
Supplement: Supplementary file 3 — (PDF 890 kb) [file 43032_2020_372_MOESM3_ESM.pdf]

S3 Figure

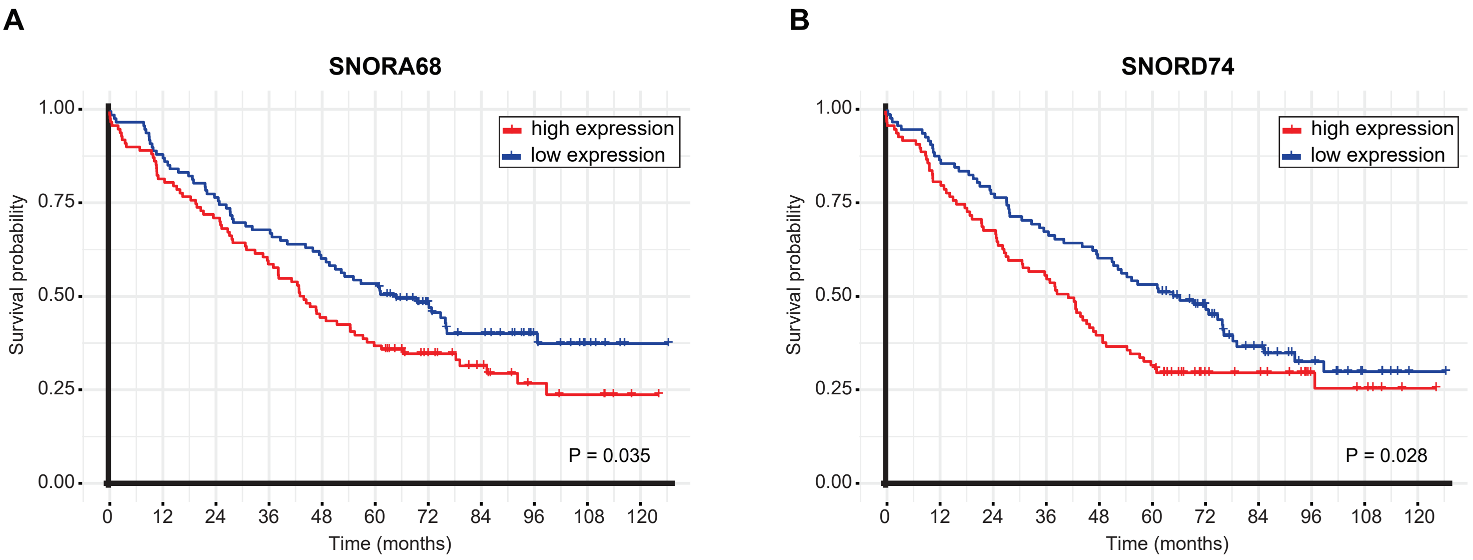

S3 Figure. Overall survival curves for SNORA68 and SNORD74 individually. “High expression” (red) and “low expression” (blue) groups represent those samples that were expressed above and below the median values for each candidate, respectively. P-values are presented above.
