# Supplementary material for: Noncoding RNA (ncRNA) Profile Association with Patient Outcome in Epithelial Ovarian Cancer Cases
Source: Reprod Sci. 2020 Oct 30;28(3):757–65. doi: 10.1007/s43032-020-00372-7 (PMC7862201; doi:10.1007/s43032-020-00372-7)
Supplement: Supplementary file 4 — (PDF 894 kb) [file 43032_2020_372_MOESM4_ESM.pdf]

S4 Figure

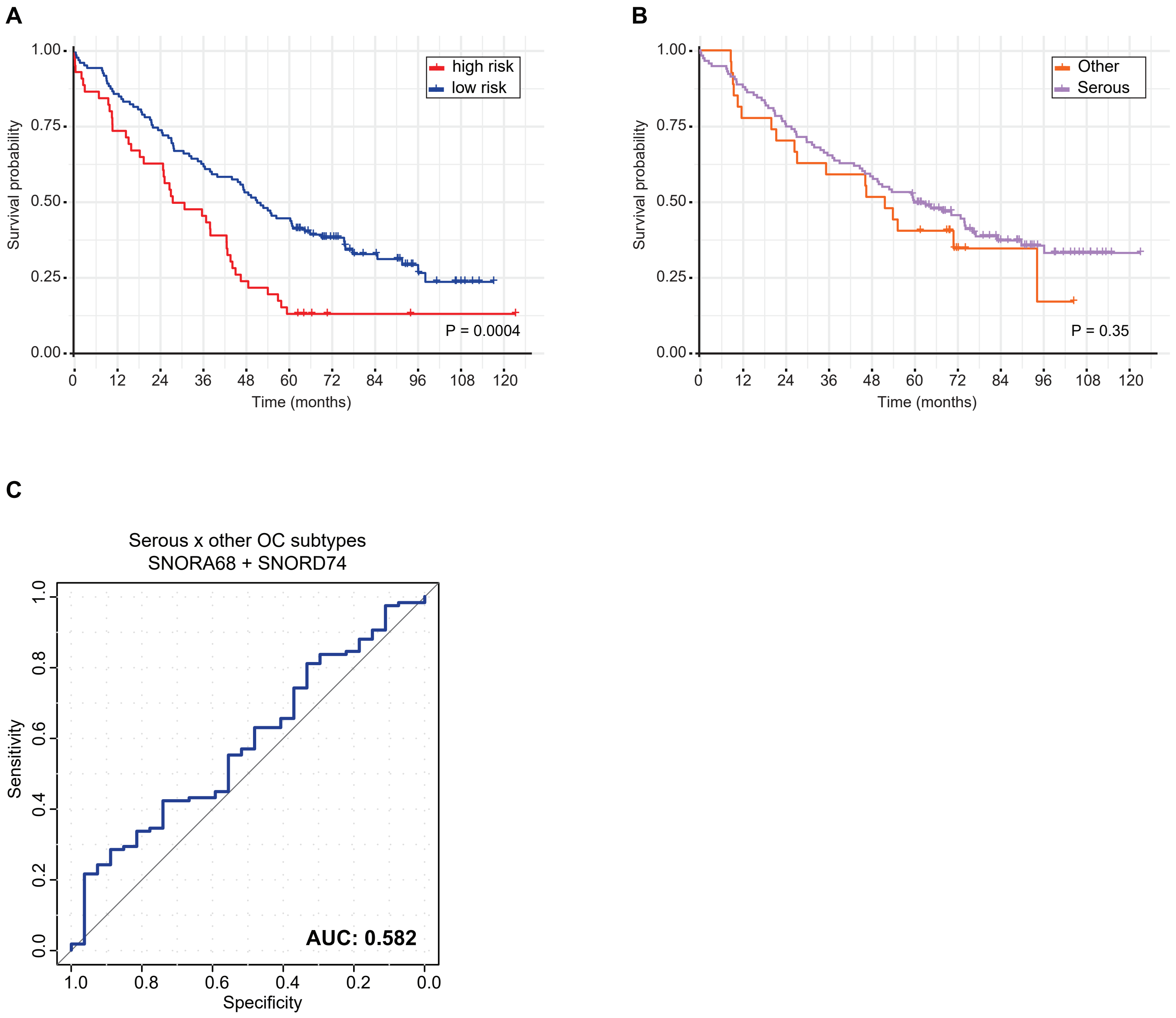

S4 Figure. (A) Survival curve between “high risk” (red) and “low risk” (blue) in serous adenocarcinoma subtype. (B) Survival curve between serous adenocarcinoma and other OC subtypes. (C) AUC/ROC curve of combination of SNORA68 and SNORD74 in serous adenocarcinoma x other OC subtypes. P-values and AUC are presented above.
