# Supplementary material for: Noncoding RNA (ncRNA) Profile Association with Patient Outcome in Epithelial Ovarian Cancer Cases
Source: Reprod Sci. 2020 Oct 30;28(3):757–65. doi: 10.1007/s43032-020-00372-7 (PMC7862201; doi:10.1007/s43032-020-00372-7)
Supplement: Supplementary file 5 — (PDF 1006 kb) [file 43032_2020_372_MOESM5_ESM.pdf]

S5 Figure

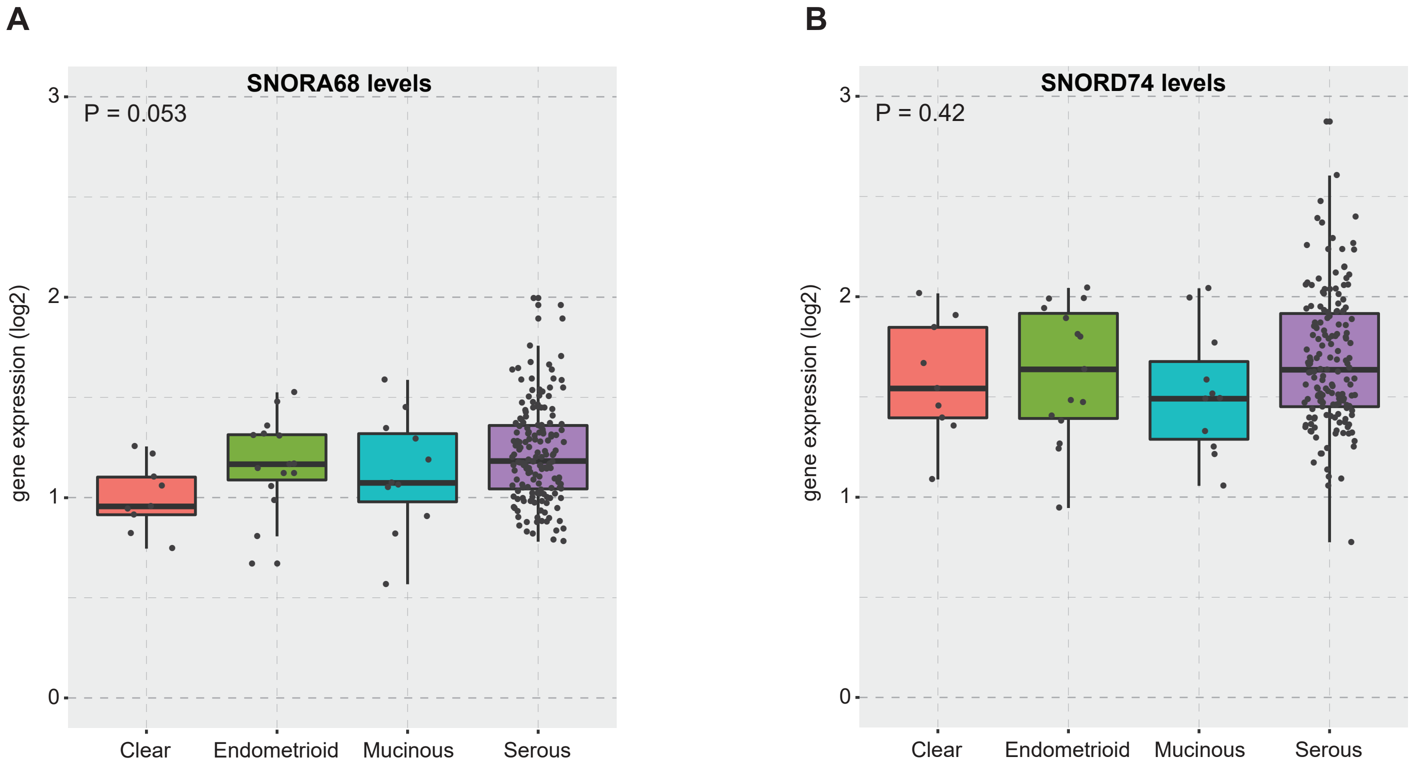

S5 Figure. Gene expression in all OC subtypes for SNORA68 (A), and SNORD74 (B). Values are normalized and provided as log_2 and P-values are presented above.
