# Supplementary material for: Noncoding RNA (ncRNA) Profile Association with Patient Outcome in Epithelial Ovarian Cancer Cases
Source: Reprod Sci. 2020 Oct 30;28(3):757–65. doi: 10.1007/s43032-020-00372-7 (PMC7862201; doi:10.1007/s43032-020-00372-7)
Supplement: Supplementary file 6 — (PDF 882 kb) [file 43032_2020_372_MOESM6_ESM.pdf]

S6 Figure

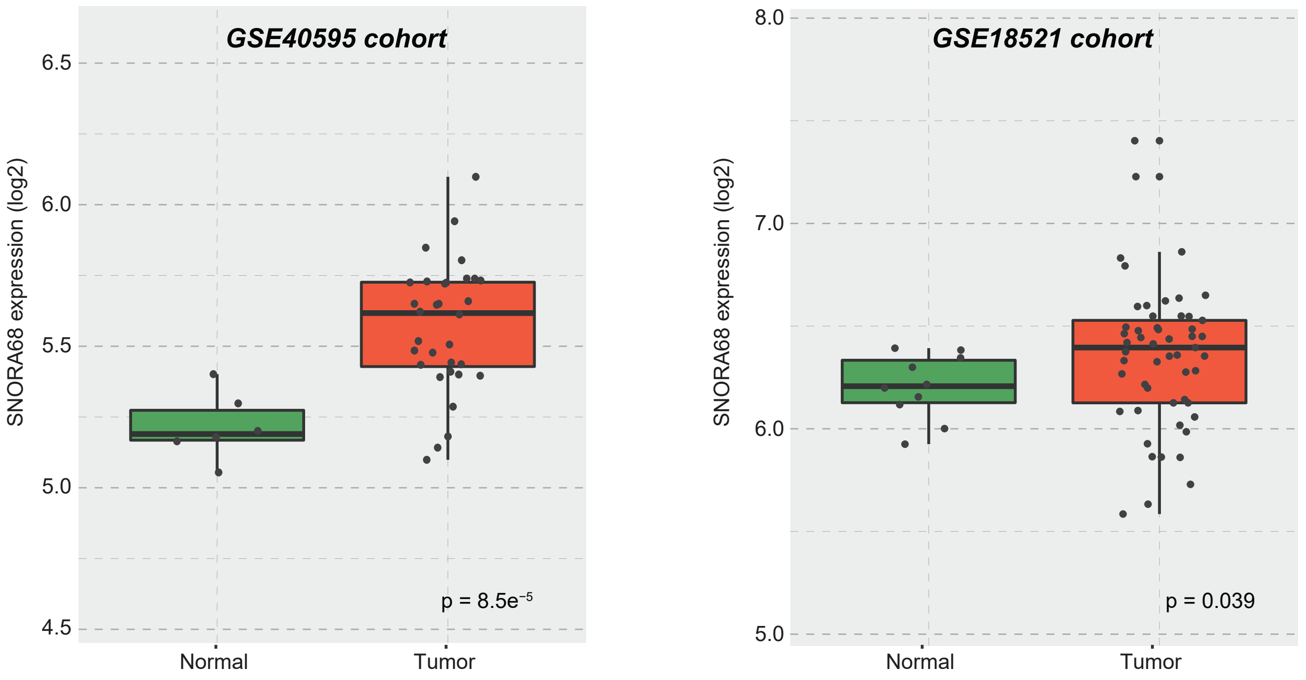

S6 Figure. SNORA68 expression in normal x OC samples. Expression values for SNORA68 in normal (green) and tumor (red) tissues in 2 independent cohorts, GSE40595 and GSE18521. Values are normalized and provided as $\log_2$ and P-value is presented above.
